# An Efficient Distributed Compressed Sensing Algorithm for Decentralized Sensor Network

**DOI:** 10.3390/s17040907

**Published:** 2017-04-20

**Authors:** Jing Liu, Kaiyu Huang, Guoxian Zhang

**Affiliations:** School of Electronics and Information Engineering, Xi’an Jiaotong University, Xi’an 710049, China; huangkaiyu@stu.xjtu.edu.cn (K.H.); zgx3135@stu.xjtu.edu.cn (G.Z.)

**Keywords:** distributed compressed sensing, JSM-1, distributed compact sensing matrix pursuit (DCSMP) algorithm

## Abstract

We consider the joint sparsity Model 1 (JSM-1) in a decentralized scenario, where a number of sensors are connected through a network and there is no fusion center. A novel algorithm, named distributed compact sensing matrix pursuit (DCSMP), is proposed to exploit the computational and communication capabilities of the sensor nodes. In contrast to the conventional distributed compressed sensing algorithms adopting a random sensing matrix, the proposed algorithm focuses on the deterministic sensing matrices built directly on the real acquisition systems. The proposed DCSMP algorithm can be divided into two independent parts, the common and innovation support set estimation processes. The goal of the common support set estimation process is to obtain an estimated common support set by fusing the candidate support set information from an individual node and its neighboring nodes. In the following innovation support set estimation process, the measurement vector is projected into a subspace that is perpendicular to the subspace spanned by the columns indexed by the estimated common support set, to remove the impact of the estimated common support set. We can then search the innovation support set using an orthogonal matching pursuit (OMP) algorithm based on the projected measurement vector and projected sensing matrix. In the proposed DCSMP algorithm, the process of estimating the common component/support set is decoupled with that of estimating the innovation component/support set. Thus, the inaccurately estimated common support set will have no impact on estimating the innovation support set. It is proven that under the condition the estimated common support set contains the true common support set, the proposed algorithm can find the true innovation set correctly. Moreover, since the innovation support set estimation process is independent of the common support set estimation process, there is no requirement for the cardinality of both sets; thus, the proposed DCSMP algorithm is capable of tackling the unknown sparsity problem successfully.

## 1. Introduction

Compressed sensing has received considerable attention recently and has been applied successfully in diverse fields, e.g., image processing [1], speech enhancement [2], sensor network [3,4] and radar systems [5]. As an important branch of compressed sensing, distributed compressed sensing (DCS) theory [6,7] rests on a new concept called the joint sparsity of a signal ensemble. A signal ensemble is composed of different signals from the various sensors of the same scene. Three joint sparsity models (JSM) are presented in [6]: JSM-1, JSM-2 and JSM-3. In JSM-1, each signal consists of a sum of two components: a common component that is present in all of the signals and an innovation component that is unique to each signal. In JSM-2, all signals are constructed from the same sparse set of basis vectors, but with different coefficient values. JSM-3 extends JSM-1 so that the common component needs no longer to be sparse in any basis. The readers can refer to [6] for more details about JSM-1, JSM-2 and JSM-3.

In this paper, we focus on the JSM-1, and such signals may arise in a sensor network where large-scale phenomena affect all sensors and local phenomena affect individual sensors. The first example would be a network of temperature sensors in a forest, where the sun has a global effect, and shade, water and animals have more local effects [6]. The second example comes from distributed spectrum sensing in cooperative cognitive networks, which consists of the estimation of a common component (due to the so-called primary users) and innovation components (due to secondary users) [8]. The third example is about joint detection and tracking of multiple targets using a multistatic radar system, where different individual receivers observe the same surveillance region with different detection probabilities. Till recently, much research work has been carried out on data aggregation, data collection and data processing in wireless sensor networks [9,10,11].

Algorithms for the distributed compressed sensing problem can be developed either in a central manner or a distributed manner [12]. The central recovery methods assume the presence of a fusion center that gathers all of the information from the network, while the decentralized recovery methods perform the reconstruction in network, with no fusion center. The decentralized recovery methods have the advantages of convenience (for implementation) and robustness (to failures), over the central ones.

Centralized reconstruction for JSM-1 has already been addressed in the literature [13,14,15,16]. In this paper, in contrast, we study a decentralized approach to JSM-1. To the best of our knowledge, there are limited literature works about the JSM-1 in a decentralized network. In [17], a distributed alternating direction method of multipliers (ADMM) is applied in the JSM-1 framework to recover both the common and the individual components. In [12], the authors develop a distributed parallel pursuit (DIPP) algorithm based on the exchange of information about estimated support sets at sensors.

In the above work, the random matrix (e.g., the random Gaussian matrix) is chosen as the sensing matrix, which provides the worst case performance guarantees in the context of an adversarial signal/error model. In this work, we focus on the deterministic sensing matrices built directly on the real acquisition systems. These kinds of matrices eliminate the need for additional measurement matrices, reduce memory storage and accelerate the reconstruction algorithm. However, the deterministic sensing matrices often encounter the high coherence problem due to the high resolution of the sensors [18]. In our previous work [19], a general similar sensing matrix pursuit (GSSMP) algorithm is proposed to cope with the high coherence problem, which obtains much better performance compared with the subspace pursuit (SP) and basis pursuit (BP) algorithms. However, the GSSMP algorithm can only tackle a single sparse signal.

In this work, the GSSMP algorithm is extended to tackle the JSM-1 in a decentralized scenario. The individual sensing matrix for each sensor is constructed based on the real acquisition system. A compact sensing matrix is built based on the original sensing matrix using similarity analysis. The compact sensing matrix has a far smaller size compared with the original sensing matrix and is proven to have low coherence. For the signal model, we use the recent proposed mixed support set model of [12], which consists of a common support set and an innovation support set. Thus, the purpose of the proposed algorithm is to estimate both the common and innovation support sets, for the reconstruction of the sparse signal of each individual sensor. The proposed algorithm is referred to the distributed compact sensing matrix pursuit (DCSMP) algorithm, and it comprises two main parts: common support set estimation and innovation support set estimation processes.

In the common support set estimation process, a candidate support set is calculated based on the compact sensing matrix and the measurement vector, for each individual sensor. We prove that the candidate support set contains the true support set. Considering that the common support set is a subset of all individual support sets of all sparse signals, we can obtain an estimated common support set by fusing the candidate support set information from an individual node and its neighboring nodes.

Next, we are to pursue the innovation support set based on the estimated common support set, for each individual sensor. The measurement vector is projected into a subspace that is perpendicular to the subspace spanned by the columns indexed by the estimated common support set, to remove the impact of it. We can then search for the innovation support set using an OMP algorithm based on the projected measurement vector and projected sensing matrix.

The main contribution of the paper has three components. First, in contrast to the conventional distributed compressed sensing algorithms adopting a random sensing matrix, the proposed DCSMP algorithm focuses on the deterministic sensing matrices built directly on the real acquisition systems, which eliminate the need for additional measurement matrices, reduce memory storage and accelerate the reconstruction algorithm. Secondly, in most algorithms addressing the JSM-1, the process of estimating the common component/support set is coupled with that of estimating the innovation component/support set; thus, the inaccurately estimated common support set will lead to failure in estimating the innovation support set. In this paper, the two processes are decoupled by projecting the measurement vector into a subspace that is perpendicular to the subspace spanned by the columns indexed by the estimated common support set, to remove the impact of it. It is proven that under the condition that the estimated common support set contains the true common support set, the proposed algorithm can find the true innovation set correctly. Thirdly, since the innovation support set estimation process is independent of the common support set estimation process, there is no requirement for the cardinality of both sets; thus, the proposed DCSMP algorithm is capable of tackling the unknown sparsity problem successfully.

The paper is organized as follows. Section 2 introduces the JSM-1 model in distributed compressed sensing. The proposed DCSMP algorithm is introduced in Section 3, which is the main contribution of this paper. The complexity and scalability analysis of the proposed DCSMP algorithm is in Section 4. In Section 5, we consider a simulation example of identifying multiple targets in a multistatic radar system, where different individual receivers observe the same surveillance region with different detection probabilities. A JSM-1 model is constructed based on the multistatic radar system, and sparse recovery is carried out in a decentralized manner across various receivers. Finally, the paper is summarized in Section 6.

### 1.1. Notation

For a set T⊂{1,2,⋯,n}, we use |T| to denote its cardinality, i.e., the number of elements in *T*. We use $T^{c}$ to denote its complement w.r.t. {1,2,⋯,n}, i.e., $T^{c}:=i\in \left\{1,2,\cdots ,n\right\}:i\notin T$.

For a vector v, v(i) denotes the *i*-th entry of v, and $v_{T}$ denotes a vector consisting of the entries of v indexed by *T*. We use ${\left||v|\right|}_{p}$ to denote the $l_{p}$ norm of v. The support set of v, supp(v), is the set of indices at which v is nonzero, supp(v):={i:v(i)≠0}. We say that v is *s*-sparse if |supp(v)|≤s.

For a matrix B, $B^{*}$ denotes its conjugate transpose and $B^{†}$ its pseudo-inverse. For a matrix with linearly-independent columns, $B^{†}={\left(B^{*}B\right)}^{-1}B^{*}$.

We use I to denote an identity matrix of appropriate size. For an index set *T* and a matrix B, $B_{T}$ is the sub-matrix of B containing columns with indices in the set *T*. Notice that $B_{T}=BI_{T}$. For a tall matrix P, span(P) denotes the subspace spanned by the column vectors of P.

## 2. Problem Formulation in the JSM-1 Framework

Consider a decentralized sensor network where a number of sensors acquire signals and communicate with neighbor nodes to reconstruct the original signals. Each individual sensor (e.g., the *p*-th sensor node) is not aware of the full network topology, and instead, it knows two sets of local neighbors; the incoming neighbor connections $L_{p}^{in}$ and outgoing neighbor connections $L_{p}^{out}$. Here, incoming and outgoing connections correspond to communication links where a node can receive or send information, respectively. In particular, it is assumed that the signals sensed by these sensors exhibit both intra-sensor correlation and inter-sensor correlation. This correlated sensing model corresponds to the JSM-1 model, which is described as follows.

The *p*-th sensor monitors a discrete signal $x_{p}\in {ℜ}^{N}$ according to the following relation:

(1) $$y_{p}=\Phi_{p}x_{p}+e_{p},\;\;\;\;\forall \;\;\;\;p\in \Gamma ,$$

where $y_{p}\in {ℜ}^{M}$ is the measurement vector, $\Phi_{p}\in {ℜ}^{M\times N}$ is the sensing matrix, $e_{p}\in {ℜ}^{M}$ is the measurement noise and Γ is a global set containing all nodes in the network. This setup describes an underdetermined system, where M<N. The signal vector $x_{p}$ is *K*-sparse, and its support set is defined as $S_{p}$. The goal of the proposed algorithm is to reconstruct the original signal observed at each sensor node, i.e., to reconstruct $x_{p}$ at the *p*-th node (p∈Γ).

In the JSM-1, each sensor has its own signal; that means signals across sensors are not the same, but have correlations. The sparse signal $x_{p}$ can be represented as:

(2) $$x_{p}=z_{c}+z_{p},$$

where $z_{c}\in {ℜ}^{N}$ denotes the common component of the sparse signal $x_{p}$, which captures the inter-signal correlation and is common to all signals, and $z_{p}\in {ℜ}^{N}\left(p\in \Gamma \right)$ denotes the innovation component of the sparse signal $x_{p}$, which captures the intra-signal correlation and is specific to the *p*-th sparse vector $x_{p}$. We further define *J* and $I_{p}$ as the support set of $z_{c}$ and $z_{p}$, respectively, and have:

(3) $$S_{p}=J\cup I_{p}\;\;\;and\;\;\;\;J⋂I_{p}=\emptyset ,\;\;\;\;\forall \;\;\;\;p\in \Gamma .$$

Here, the partial support set *J* is common to all sparse signals, which is defined as the common support set. The partial support set $I_{p}$ is specific for the *p*-th node and defined as the innovation support set.

## 3. Distributed Compact Sensing Matrix Pursuit Algorithm

**Figure 1:**
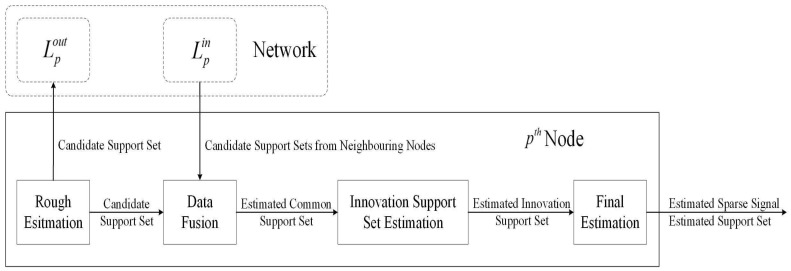
Block diagram of the DCSMP algorithm.

The goal of this section is to introduce the proposed DCSMP algorithm. A block diagram of the DCSMP algorithm is shown in Figure 1, which comprises four stages: (1) rough estimation, (2) data fusion, (3) innovation support set estimation and (4) final estimation. The estimated common support set is generated at the first and second stage; the estimated innovation support set is obtained at the third stage; and finally, we can obtain the final estimates of the support set and the original sparse signal at the fourth stage.

### 3.1. Rough Estimation

The goal of this section is to calculate the candidate support set for each individual sensor. In order to tackle the high coherence problem due to the high resolution of the sensors, we consider constructing a compact sensing matrix for each individual sensing matrix at each node, which is a compact version of the original sensing matrix and has low coherence. The readers can refer to [19] for details of the construction process of the compact sensing matrix. An OMP algorithm is then utilized to calculate a rough estimate of the true support set, based on which we can obtain the candidate support set ${\hat{F}}_{p}$ in the original sensing matrix. Finally, we prove that the candidate support set contains the true support set for each individual sensor. The detailed procedures of the candidate support set estimation (CSSE) algorithm are presented in Algorithm 1.

 **Algorithm 1:** Candidate support set estimation (CSSE).
**Input**: $\Phi_{p}$, $y_{p}$
**Output**: ${\hat{F}}_{p}$  1.$\Psi_{p}$ ← ConstructCompact($\Phi_{p}$).2.${\hat{a}}_{p}$ ← OMP($\Psi_{p}$, $y_{p}$).3.${\hat{\Xi }}_{p}^{ini}$ ← MapToSubspace(${\hat{a}}_{p}$).4.${\hat{F}}_{p}$ ← FindCandidateSupportSet (${\hat{\Xi }}_{p}^{ini}$).

The candidate support set estimation algorithm consists of four steps. The first step uses the “ConstructCompact” function, which constructs the compact sensing matrix $\Psi_{p}$ based on the original sensing matrix $\Phi_{p}$. The process is the same as that described in Section 2.3 in [19]. At the second step, the OMP algorithm is used to find an estimate of the true support set, which is represented as ${\hat{a}}_{p}=\left\{{\hat{a}}_{p}^{1},\cdots ,{\hat{a}}_{p}^{k},\cdots ,{\hat{a}}_{p}^{K^{\prime }}\right\}$, $K^{\prime }\leq K$, where ${\hat{a}}_{p}^{k}$ denotes the *k*-th element of ${\hat{a}}_{p}$. At the third step, in the “MapToSubspace” function, each element in the estimated support set ${\hat{a}}_{p}^{k}$ corresponds to a condensed column of the compact sensing matrix. For example, ${\hat{a}}_{p}^{k}\left(\beta_{p}^{j}\right)$ indicates that the *k*-th element of ${\hat{a}}_{p}$ corresponds to the *j*-th condensed column $\beta_{p}^{j}$. This column is defined as a contributing column. We can then obtain an initial estimate of the correct subspace, ${\hat{\Xi }}_{p}^{ini}$, spanned by $K^{\prime }$ contributing columns $\left\{\beta_{p}^{i},\cdots ,\beta_{p}^{j},\cdots ,\beta_{p}^{l}\right\}$, as ${\hat{\Xi }}_{p}^{ini}=$ span$\left(\beta_{p}^{i},\cdots ,\beta_{p}^{j},\cdots ,\beta_{p}^{l}\right)$. The final step uses the function “FindCandidateSupportSet”, which is to find the candidate support set in the original sensing matrix $\Phi_{p}$. Each contributing column corresponds to a similar column group in $\Phi_{p}$, which is named the contributing similar column group. We can then obtain a set ${\hat{\Lambda }}_{p}$ containing the indices of $K^{\prime }$ contributing similar column groups. All of the columns in each contributing similar column group from ${\hat{\Lambda }}_{p}$ are listed out, and their indices form a candidate support set ${\hat{F}}_{p}$.

**Proposition** **1.** *The true support set* $S_{p}$ *is a subset of the candidate support set* ${\hat{F}}_{p}$*, i.e.,* $S_{p}\subset {\hat{F}}_{p}$.

**Proof.**  According to Proposition 2 in [19], the vectors spanning the true subspace are contained in $K^{\prime }$ contributing similar column groups. Thus, the true support set is contained in the candidate support set ${\hat{F}}_{p}$, which is the union of the indices of the columns contained in the $K^{\prime }$ contributing similar column groups. ☐

At the end of the rough estimation stage, the *p*-th node sends its own candidate support set to its neighboring nodes, as well as receives the candidate support sets from its neighboring nodes. The candidate support sets from both the *p*-th node and its neighboring nodes are sent to the data fusion stage, where the estimated common support set is generated by using some fusion strategy.

### 3.2. Data Fusion

For the *p*-th node, its true support set $S_{p}$ (including the common support set *J* and innovation support set $I_{p}$) is contained in the candidate support set ${\hat{F}}_{p}$, according to Proposition 1. Considering that the common support set is joint to the *p*-th node and its connected neighboring nodes, it can be obtained by fusing the candidate support sets from both the *p*-th node and its neighboring nodes in the network. For fusion, we use a democratic voting strategy [12].

The data fusion algorithm is presented in Algorithm 2. The *p*-th node has access to the candidate support sets ${\left\{{\hat{F}}_{q}\right\}}_{q\in L_{p}^{in}}$ from neighbors and the local estimate ${\hat{F}}_{p}$. The estimated common support set $\hat{J}$ is formed (Step 5) such that each index in the estimated common support set is present in at least two candidate support sets from $\left\{{\left\{{\hat{F}}_{q}\right\}}_{q\in L_{p}^{in}},{\hat{F}}_{p}\right\}$. Having more votes for a certain index increases the probability of this index being correct.

 **Algorithm 2:** Data fusion.  Input: ${\left\{{\hat{F}}_{q}\right\}}_{q\in L_{p}^{in}}$, ${\hat{F}}_{p}$  Output: $\hat{J}$  Initialization: $z\leftarrow 0_{N\times 1}$  1: $z\leftarrow vote_{1}\left(z,{\hat{F}}_{p}\right)$  2: for each $q\in L_{p}^{in}$ do  3: $\;\;\;\;z\leftarrow vote_{1}\left(z,{\hat{F}}_{q}\right)$  4: end for   5: Choose $\hat{J}$ s.t. $\left(z\left(i\right)\geq 2\right)\;\;\forall \;i\in \hat{J}$

In the above algorithm, “$vote_{1}$” denotes the voting procedure [12]. Since an index present in two nodes’ candidate support sets will be treated as an element of the estimated common support set, the probability of the event that the estimated common support set contains the true common support set is very high. Thus, it is reasonable to assume that the true common support set is a subset of the estimated common support set.

### 3.3. Innovation Support Set Estimation

We consider a general condition that the true common support set is a subset of the estimated common support set. The related assumptions are listed as follows.

**Assumption** **A1.** *The true common support set J and the innovation support set* $I_{p}$*, satisfy the following conditions:*

1. *The true common support set J is a subset of the estimated common support set* $\hat{J}$*, i.e.,* $J\subseteq \hat{J}$.
2. *The innovation support set* $I_{p}$ *is a proper subset of the complement of* $\hat{J}$*, i.e.,* $I_{p}\subset {\hat{J}}^{c}$.

The second assumption holds considering that the cardinality of $I_{p}$ is far less than that of ${\hat{J}}^{c}$.

The goal of the innovation support set estimation stage is to calculate $I_{p}$. We prove Lemma 1 before we proceed to the detailed procedures of the proposed algorithm.

**Lemma** **1.** *Assuming that* $|\hat{J}|\leq 2K$*, define the projection matrix* $P_{p}$ *as:*

(4) $$P_{p}=I-{\left(\Phi_{p}\right)}_{\hat{J}}{\left[{\left(\Phi_{p}\right)}_{\hat{J}}^{*}{\left(\Phi_{p}\right)}_{\hat{J}}\right]}^{-1}{\left(\Phi_{p}\right)}_{\hat{J}}^{*},$$

*and the projected measurement vector* ${\tilde{y}}_{p}$ *as:*

(5) $${\tilde{y}}_{p}=P_{p}y_{p}.$$

*The projected measurement vector* ${\tilde{y}}_{p}$ *can be represented in a standard equation in compressed sensing as:*

(6) $$\begin{array}{c} {\tilde{y}}_{p}=A_{p}{\left(x_{p}\right)}_{{\hat{J}}^{c}}+e_{p}^{\prime }, \end{array}$$

*where* $A_{p}=P_{p}{\left(\Phi_{p}\right)}_{{\hat{J}}^{c}}$ *is the projected sensing matrix and* $e_{p}^{\prime }=P_{p}e_{p}$ *is the equivalent noise. Moreover, the innovation support set* $I_{p}$ *can be calculated using an OMP algorithm, based on the projected measurement vector* ${\tilde{y}}_{p}$ *and projected sensing matrix* $A_{p}$.

**Proof.**  The proof consists of three parts. First, we prove that ${\left(\Phi_{p}\right)}_{\hat{J}}$ has full column rank under the condition that the cardinality of the estimated common support set $\hat{J}$ is less than or equal to 2K, i.e., $|\hat{J}|\leq 2K$, where *K* is the sparsity level of $x_{p}$.

According to Theorem 2.13 in [20], a unique *s*-sparse solution of the system y=Ax implies that every set of 2s columns of A is linearly independent, where *s* indicates the sparsity level of x. Theorem 2.13 applies to the deterministic sensing matrix in this work, since the system has a unique *K*-sparse solution. Thus, every set of 2K columns of $\Phi_{p}$ is linearly independent. Under the condition that $|\hat{J}|\leq 2K$, the submatrix ${\left(\Phi_{p}\right)}_{\hat{J}}$ contains at most 2K columns. Since every set of 2K columns of $\Phi_{p}$ is linearly independent, ${\left(\Phi_{p}\right)}_{\hat{J}}$ has full column rank.

Secondly, we prove that the projected measurement vector ${\tilde{y}}_{p}$ can be represented in a standard equation in compressed sensing.

(7) $$\begin{array}{ccc} {\tilde{y}}_{p} & = & P_{p}y_{p}\\ & = & P_{p}\left(\Phi_{p}x_{p}+e_{p}\right)\\ & = & P_{p}\left[{\left(\Phi_{p}\right)}_{\hat{J}}{\left(x_{p}\right)}_{\hat{J}}+{\left(\Phi_{p}\right)}_{{\hat{J}}^{c}}{\left(x_{p}\right)}_{{\hat{J}}^{c}}+e_{p}\right]\\ & = & P_{p}{\left(\Phi_{p}\right)}_{{\hat{J}}^{c}}{\left(x_{p}\right)}_{{\hat{J}}^{c}}+P_{p}e_{p},\\ & = & A_{p}{\left(x_{p}\right)}_{{\hat{J}}^{c}}+e_{p}^{\prime } \end{array}$$

The fourth equality of (7) holds considering that the contribution of $\hat{J}$ to $y_{p}$ can be nullified, by projecting $y_{p}$ into a perpendicular space using the projection matrix $P_{p}$.

(8) $$\begin{array}{cc} & P_{p}{\left(\Phi_{p}\right)}_{\hat{J}}{\left(x_{p}\right)}_{\hat{J}}\\ = & \left\{I-{\left(\Phi_{p}\right)}_{\hat{J}}{\left[{\left(\Phi_{p}\right)}_{\hat{J}}^{*}{\left(\Phi_{p}\right)}_{\hat{J}}\right]}^{-1}{\left(\Phi_{p}\right)}_{\hat{J}}^{*}\right\}{\left(\Phi_{p}\right)}_{\hat{J}}{\left(x_{p}\right)}_{\hat{J}}\\ = & 0 \end{array}$$

Thus, we can obtain (6), a standard equation in compressed sensing.

Finally, we will prove that $I_{p}$ is the support set of the sparse vector ${\left(x_{p}\right)}_{{\hat{J}}^{c}}$. The support set of $x_{p}$ can be represented as:

(9) $$S_{p}=J\cup I_{p}\;\;\;and\;\;\;\;J⋂I_{p}=\emptyset ,\;\;\;\;\forall \;\;\;\;p\in \Gamma .$$

According to Assumption 1, *J* is a subset of $\hat{J}$ and, thus, is the set of indices at which ${\left(x_{p}\right)}_{\hat{J}}$ is nonzero. Similarly, $I_{p}$ is the proper subset of ${\hat{J}}^{c}$ and is the set of indices at which ${\left(x_{p}\right)}_{{\hat{J}}^{c}}$ is nonzero. Thus, $I_{p}$ is the support set of the sparse vector ${\left(x_{p}\right)}_{{\hat{J}}^{c}}$. ☐

In summary, (6) is a standard equation in compressed sensing, and we can calculate $I_{p}$ using an OMP algorithm based on the projected measurement vector ${\tilde{y}}_{p}$ and projected sensing matrix $A_{p}$.

 **Algorithm 3:** Innovation support set estimation (ISSE).
**Input**: $\Phi_{p}$, $y_{p}$, $\hat{J}$
**Output**: ${\hat{I}}_{p}$  1.$P_{p}$←$I-{\left(\Phi_{p}\right)}_{\hat{J}}{\left[{\left(\Phi_{p}\right)}_{\hat{J}}^{*}{\left(\Phi_{p}\right)}_{\hat{J}}\right]}^{-1}{\left(\Phi_{p}\right)}_{\hat{J}}^{*}$.2.${\tilde{y}}_{p}$←$P_{p}y_{p}$.3.$A_{p}$←$P_{p}{\left(\Phi_{p}\right)}_{{\hat{J}}^{c}}$.4.${\hat{I}}_{p}$← OMP(${\tilde{y}}_{p}$, $A_{p}$).

In the above algorithm (Algorithm 3), the first step is to construct the projection matrix $P_{p}$, while the second and third steps are to construct the projected measurement vector ${\tilde{y}}_{p}$ and projected sensing matrix $A_{p}$. Finally, the innovation support set ${\hat{I}}_{p}$ can be calculated using an OMP algorithm, based on ${\tilde{y}}_{p}$ and $A_{p}$.

### 3.4. Final Estimation

The goal of this section is to estimate the support set $S_{p}$ and the sparse vector $x_{p}$, given the estimated common support set $\hat{J}$ and the estimated innovation support set ${\hat{I}}_{p}$. First, a combinational search is performed in $\hat{J}$ to find the true common support set *J*. Each combination together with ${\hat{I}}_{p}$ forms an estimate of the true support set, which is denoted as ${\hat{S}}_{p}^{j},\;j=1,2,\cdots ,N_{co}$, where $N_{co}$ indicates the number of combinations. Based on each estimated support set, we can obtain an estimate of the sparse vector $x_{p}$ (denoted as ${\hat{x}}_{p}^{j}$), using the pseudo-inverse operation. Among the obtained estimated sparse vectors ${\hat{x}}_{p}^{j},\;j=1,2,\cdots ,N_{co}$, we can find the one with the least residual, which is termed as the final estimate of the sparse vector $x_{p}$. The detailed procedures are as follows.

The combinational search algorithm consists of six steps. The first step uses the “ListCombinations” function, which lists $C_{E}^{1},C_{E}^{2},\cdots ,C_{E}^{E}$ combinations based on the indices in $\hat{J}$, where $E=|\hat{J}|$. Each combination is represented as $JI^{j},\;j=1,2,\cdots ,N_{co}$. At the second step, each combination together with ${\hat{I}}_{p}$ forms an estimate of the true support set, i.e., ${\hat{S}}_{p}^{j}=JI^{j}⋃{\hat{I}}_{p},\;j=1,2,\cdots ,N_{co}$. At the third step, the proposed algorithm solves a least squares problem to approximate the nonzero entries (${\left({\hat{x}}_{p}^{j}\right)}_{{\hat{S}}_{p}^{j}}$←${\left[{\left(\Phi_{p}\right)}_{{\hat{S}}_{p}^{j}}\right]}^{†}\;y_{p}$) and sets other entries as zero (${\left({\hat{x}}_{p}^{j}\right)}_{{\left({\hat{S}}_{p}^{j}\right)}^{c}}$←0), resulting in an estimate of the sparse vector, ${\hat{x}}_{p}^{j},\;j=1,2,\cdots ,N_{co}$. The fourth step is to calculate the residual $r_{p}^{j}\;\left(j=1,\cdots ,N_{co}\right)$. The $l_{2}$ norm of $r_{p}^{j}$ is indicated as $\left|\right|r_{p}^{j}{\left|\right|}_{2}$. The fifth step uses the “MinimumResidual” function, which finds the residual with the least $l_{2}$ norm among the residuals, and denotes it as $r_{p}^{min}$. Concurrently, we can find its associate sparse signal and support set, denoted as ${\hat{x}}_{p}^{min}$ and ${\hat{S}}_{p}^{min}$, respectively. Finally, we can obtain the final estimates of the sparse signal and the true support set, by setting ${\hat{x}}_{p}$←${\hat{x}}_{p}^{min}$ and ${\hat{S}}_{p}$←${\hat{S}}_{p}^{min}$.

**Proposition** **2.** *The proposed combinational search algorithm (Algorithm 4) can find the true support set* $S_{p}$*, provided that* ${\hat{I}}_{p}$ *is correctly estimated.*

**Proof.**  In Algorithm 4, $C_{E}^{1},C_{E}^{2},\cdots ,C_{E}^{E}$ combinations are listed based on the indices in $\hat{J}$, where $E\;=\;|\hat{J}|$. Each combination is represented as $JI^{j},\;j=1,2,\cdots ,N_{co}$, where $N_{co}$ indicates the number of combinations. Since $J\subseteq \hat{J}$, *J* is contained in $JI^{j},\;j=1,2,\cdots ,N_{co}$. Thus, $S_{p}=J⋃I_{p}$ is contained in $JI^{j}⋃{\hat{I}}_{p}$ provided that ${\hat{I}}_{p}$ is correctly estimated. Therefore, we can find the true support set $S_{p}$ via the combinational search algorithm. ☐

**Algorithm 4:** Combinational search.
**Input**: $\Phi_{p}$, $y_{p}$, $\hat{J}$, ${\hat{I}}_{p}$
**Output**: ${\hat{S}}_{p}$, ${\hat{x}}_{p}$  1.$JI^{j}$ ← ListCombinations ($\hat{J}$), $j=1,2,\cdots ,N_{co}$.2.${\hat{S}}_{p}^{j}$←$JI^{j}⋃{\hat{I}}_{p}$, $j=1,2,\cdots ,N_{co}$.3.${\left({\hat{x}}_{p}^{j}\right)}_{{\hat{S}}_{p}^{j}}$←${\left[{\left(\Phi_{p}\right)}_{{\hat{S}}_{p}^{j}}\right]}^{†}\;y_{p}$, ${\left({\hat{x}}_{p}^{j}\right)}_{{\left({\hat{S}}_{p}^{j}\right)}^{c}}$←0, $j=1,2,\cdots ,N_{co}$.4.$r_{p}^{j}$←$y_{p}-\Phi_{p}{\hat{x}}_{p}^{j}$, $j=1,2,\cdots ,N_{co}$.5.$r_{p}^{min}$← MinimumResidual ($\left\{r_{p}^{1},r_{p}^{2},\cdots ,r_{p}^{N_{co}}\right\}$). Concurrently, find ${\hat{x}}_{p}^{min}$ and ${\hat{S}}_{p}^{min}$.6.${\hat{x}}_{p}$←${\hat{x}}_{p}^{min}$, ${\hat{S}}_{p}$←${\hat{S}}_{p}^{min}$.

### 3.5. DCSMP Algorithm

Using Algorithms 1∼4, we now develop the DCSMP algorithm presented in Algorithm 5. The inputs to Algorithm 5 for the *p*-th node are the measurement signal $y_{p}$ and the sensing matrix $\Phi_{p}$. Furthermore, Algorithm 5 knows $L_{p}^{in}$ and $L_{p}^{out}$. We assume that some underlying communication scheme is provided for the transmit and receive functionality.

In Algorithm 5, local candidate support set estimates are exchanged over the network (Steps 2 and 3). The data fusion algorithm merges the local and neighboring candidate support set estimates to produce the estimated common support set $\hat{J}$ (Step 4). At Step 5, the contribution of $\hat{J}$ to $y_{p}$ is nullified, by projecting $y_{p}$ into a subspace perpendicular to the space spanned by the columns of $\Phi_{p}$, indexed by $\hat{J}$. We can then calculate the estimated innovation support set ${\hat{I}}_{p}$ using an OMP algorithm based on the projected measurement vector and projected sensing matrix. Finally, a combinational search is performed in $\hat{J}$ to find *J*, and thus, we can obtain the final estimates of the support set and the original sparse vector.

 **Algorithm 5:** Distributed CSMP.
**Input**: $y_{p}$, $\Phi_{p}$, $L_{p}^{in}$, $L_{p}^{out}$
**Output**: ${\hat{S}}_{p},{\hat{x}}_{p}$  1.${\hat{F}}_{p}$ ← CSSE($\Phi_{p}$, $y_{p}$);2.Transmit: send ${\hat{F}}_{p}$ to its neighboring nodes;3.Receive: Receive ${\left\{{\hat{F}}_{q}\right\}}_{q\in L_{p}^{in}}$ from the neighboring nodes;4.$\hat{J}$ ← DataFusion(${\left\{{\hat{F}}_{q}\right\}}_{q\in L_{p}^{in}}$, ${\hat{F}}_{p}$);5.${\hat{I}}_{p}$ ← ISSE($\Phi_{p}$, $y_{p}$, $\hat{J}$);6.(${\hat{S}}_{p}$, ${\hat{x}}_{p}$) ← CombinationalSearch($\Phi_{p}$, $y_{p}$, $\hat{J}$, ${\hat{I}}_{p}$)

Discussion: Two additional settings are considered when information from all sensors can be fused. The first setting is that each sensor has the capability of communicating with all other sensors in the network, and the second one is that the measurements of all sensors are sent to a fusion center, which generates an estimate of the original signal using information from all sensors.

It is straightforward to extend the proposed DCSMP algorithm to the above two settings. To deal with the first condition, we need only change the inputs of the data fusion algorithm (Algorithm 2). For instance, for the *p*-th node, change the original inputs of the data fusion algorithm, the candidate support sets from its neighbors, ${\left\{{\hat{F}}_{q}\right\}}_{q\in L_{p}^{in}}$, to the candidate support sets from all other sensors in the network, ${\left\{{\hat{F}}_{q}\right\}}_{q\neq p}$. For the second one, when the measurements from all of the sensors in the network are sent to the fusion center, we need only change the inputs of the data fusion algorithm (Algorithm 2) to the candidate support sets from all of the sensors in the network.

## 4. Complexity and Scalability Analysis

### 4.1. Complexity Analysis

The proposed DCSMP algorithm consists of two main parts: offline processing and online processing. The offline processing transforms the individual sensing matrix $\Phi_{p}$ to a compact sensing matrix $\Psi_{p}$ using similarity analysis. The computational complexity of the offline processing mainly focuses on the computation of the similarity between any two columns of the individual sensing matrix, which is of the order of O$\left(\frac{N(N-1)}{2}M\right)$ [19]. *M* and *N* are the numbers of the rows and columns of $\Phi_{p}$, respectively.

The online processing procedure consists of four parts: (1) rough estimation; (2) data fusion; (3) innovation support set estimation; and (4) final estimation. First, for the rough estimation process, an OMP algorithm is used to find a rough estimate of the true support set for each individual signal. The computational complexity is of the order of O($MD_{p}$) for the *p*-th node, where $D_{p}$ is the number of columns of the compact sensing matrix $\Psi_{p}$ [19].

Secondly, for the data fusion process, we use a democratic voting strategy [12], which is very simple and has negligible complexity compared with the other three processes.

In the innovation support set estimation process, first, the measurement vector $y_{p}$ is projected into the subspace that is perpendicular to the space spanned by the columns of $\Phi_{p}$, which are indexed by the estimated common support set $\hat{J}$, as:

(10) $$\begin{array}{ccc} {\tilde{y}}_{p} & = & P_{p}y_{p}\\ & = & \left\{I-{\left(\Phi_{p}\right)}_{\hat{J}}{\left[{\left(\Phi_{p}\right)}_{\hat{J}}^{*}{\left(\Phi_{p}\right)}_{\hat{J}}\right]}^{-1}{\left(\Phi_{p}\right)}_{\hat{J}}^{*}\right\}y_{p}\\ & = & y_{p}-{\left(\Phi_{p}\right)}_{\hat{J}}{\left(\Phi_{p}\right)}_{\hat{J}}^{†}y_{p}. \end{array}$$

The complexity of this step concentrates on the pseudo-inverse operation, i.e., computing ${\left(\Phi_{p}\right)}_{\hat{J}}^{†}y_{p}$, using the least squares algorithm. Thus, the computational cost focuses on the least squares estimation and is of the order of O($|\hat{J}|\cdot M$), according to [21]. Secondly, the estimated innovation support set ${\hat{I}}_{p}$ is calculated using an OMP algorithm, and the complexity of this step is O($M\cdot |{\hat{J}}^{c}|$). Thus, the complexity of entire innovation support set estimation is O($|\hat{J}|\cdot M$)+O($M\cdot |{\hat{J}}^{c}|$).

In the final estimation process, $C_{E}^{1},C_{E}^{2},\cdots ,C_{E}^{E}$ combinations are listed out based on the indices in $\hat{J}$, where $E=|\hat{J}|$. Each combination is represented as $JI^{j},\;j=1,2,\cdots ,N_{co}$, where $N_{co}=C_{E}^{1}+C_{E}^{2}+\cdots +C_{E}^{E}$. Each combination together with ${\hat{I}}_{p}$ forms an estimate of the true support set, i.e., ${\hat{S}}_{p}^{j}=JI^{j}⋃{\hat{I}}_{p},\;j=1,2,\cdots ,N_{co}$. Based on each estimated support set, the nonzero entries of the estimated sparse vector are calculated using the least squares algorithm. The computational cost of the final estimation process is of the order of $C_{E}^{1}\cdot O(|{\hat{S}}_{p}^{1}|\cdot M)+C_{E}^{2}\cdot O(|{\hat{S}}_{p}^{2}|\cdot M)+\cdots +C_{E}^{E}\cdot O(|{\hat{S}}_{p}^{E}\left|\cdot M\right)$ [21]. Furthermore, since $max(|{\hat{S}}_{p}^{1}|,|{\hat{S}}_{p}^{2}|,\cdots ,|{\hat{S}}_{p}^{E}|)\leq K$, we have:

(11) $$\begin{array}{ccc} C_{E}^{1}\cdot O(|{\hat{S}}_{p}^{1}|\cdot M)+\cdots +C_{E}^{E}\cdot O(|{\hat{S}}_{p}^{E}\left|\cdot M\right) & < & C_{E}^{1}\cdot O\left(KM\right)+\cdots +C_{E}^{E}\cdot O\left(KM\right) \end{array}$$

Considering that $C_{E}^{1}\cdot O\left(KM\right)+\cdots +C_{E}^{E}\cdot O\left(KM\right)=N_{co}\cdot O\left(KM\right)$, the computational cost of the final estimation process can be approximated as the order of $N_{co}\cdot O\left(KM\right)$.

In summary, the complexity analysis for online processing is listed in Table 1, and the computational complexity of the whole DCSMP algorithm is listed in Table 2.

### 4.2. Scalability Analysis

In a typical wireless sensor network (WSN) scenario, signals are sampled at source nodes and aggregated at sink nodes. The correlation between source nodes causes redundancy. Many methods are proposed to reduce the redundancy, such as the structure fidelity data collection approach [11] and distributed compressed sensing [22].

This paper considers a decentralized sensor network where a number of densely-placed sensors acquire signals and communicates with neighbor nodes to reconstruct the original signals. All of the sensor nodes are equivalent. Each individual sensor (e.g., the *p*-th sensor node) is not aware of the full network topology, and instead, it knows two sets of local neighbors: the incoming neighbor connections $L_{p}^{in}$ and outgoing neighbor connections $L_{p}^{out}$. Here, incoming and outgoing connections correspond to communication links where a node can receive or send information, respectively. The sparse signal is sampled and reconstructed at each sensor node, by exploiting the correlation between the sensor nodes. In particular, at the *p*-th node, the DCSMP algorithm fuses the candidate support set estimates from both the *p*-th node and its neighboring nodes, to enhance the reconstruction performance.

In the conventional sensor network, as the size of the network expands, the transmission burden and the computational cost of the data fusion process increase dramatically at each node. However, in the DCSMP algorithm-based sensor network, the candidate support sets are transmitted over the network, rather than the transmitted measurements as in the conventional network. This significantly reduces the transmission burden on the links. Moreover, the computational cost of the data fusion process grows linearly with the number of incoming neighboring connections. The computational complexity of the data fusion process is negligible since a very simple democratic voting strategy [12] is adopted in this work. Thus, the DCSMP algorithm provides a desirable structural scalability.

## 5. Simulation Results and Analysis

**Figure 2:**
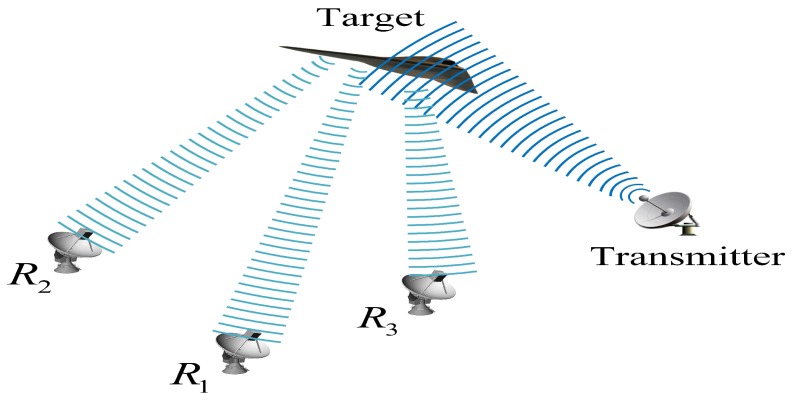
Multistatic radar system.

In this section, we consider a distributed multistatic radar system, which consists of a transmitter and a number of receivers (Figure 2). The transmitter emits the transmitted signal; the receivers receive the echoes from the targets. It is assumed that different individual receivers observe a same surveillance region with different detection probabilities. A JSM-1 is constructed based on the multistatic radar system, and sparse recovery is carried out in a decentralized manner across various receivers.

### 5.1. Sparse Representation in State Space

We consider $N_{T}$ targets moving within the surveillance region. The state vector of the *d*-th target $\left(d=1,\cdots ,N_{T}\right)$ at the *k*-th scan is defined as $x_{k}^{d}={\left[px_{k}^{d},vx_{k}^{d},py_{k}^{d},vy_{k}^{d},pz_{k}^{d},vz_{k}^{d}\right]}^{T}$, where $px_{k}^{d}$ and $vx_{k}^{d}$ denote respectively the position and velocity of the *d*-th target along the *x* axis of Cartesian frame at scan *k*; $py_{k}^{d}$ and $vy_{k}^{d}$ along the *y* axis and $pz_{k}^{d}$ and $vz_{k}^{d}$ along the *z* axis.

In the multistatic radar system, a transmitter Tr is at a known position $\mathrm{tr}={\left[x_{0},y_{0},z_{0}\right]}^{T}$; a number of receivers $R_{i}$ ($i=1,\cdots ,N_{R}$) are placed at known locations $r_{i}={\left[x_{i},y_{i},z_{i}\right]}^{T},i=1,\cdots ,N_{R}$, in a Cartesian coordinate system, where $N_{R}$ denotes the number of receivers.

In practice, the number, locations and velocities of the targets are unknown during the tracking process. The state space at scan *k* is divided into $N_{g}$ grids (possible values), listed as $g_{k}^{l},\;l=1,\cdots ,N_{g}$. An auxiliary parameter, namely grid reflection, is attached to each grid. If a grid is occupied by a target, its grid reflection parameter is set as the reflection coefficient of the target; otherwise, it is set as zero. All of the grid reflection parameters are mapped into a grid reflection vector $\xi_{k}$, which is an indicator vector that contains the true reflectivity of targets at each grid location. Considering that the number of grids occupied by targets is much smaller than that of the total grids in state space, $\xi_{k}$ is a sparse vector.

Each grid in the state space represents a state vector of a potential target. The *l*-th grid $g_{k}^{l}$ is transformed to a delay-Doppler set $\left(\tau_{k}^{i,l},f_{k}^{i,l}\right)$, according to Equations (12) and (13), under the condition of receiver $R_{i}$,

(12) $$\tau_{k}^{i,l}=\frac{1}{c}\left(\left|\right|P_{k}^{l}-\mathrm{tr}||+\left|\right|P_{k}^{l}-r_{i}\left|\right|\right),$$

(13) $$f_{k}^{i,l}=\frac{f_{c}}{c}\left(\langle V_{k}^{l},u_{k}^{i,l}\rangle -\langle V_{k}^{l},u_{k}^{tr,l}\rangle \right),$$

where $P_{k}^{l}={\left[px_{k}^{l},py_{k}^{l},pz_{k}^{l}\right]}^{T}$ and $V_{k}^{l}={\left[vx_{k}^{l},vy_{k}^{l},vz_{k}^{l}\right]}^{T}$ denote the position and velocity of the *l*-th grid at scan *k*, respectively; $u_{k}^{tr,l}$ and $u_{k}^{i,l}$ denote the unit vector from the transmitter to the *l*-th grid and the unit vector from the *l*-th grid to the *i*-th receiver, respectively.

Note: From (12) and (13), it can be seen that a grid in the state space, $g_{k}^{l}$, corresponds to different delay-Doppler set $\left(\tau_{k}^{i,l},f_{k}^{i,l}\right)$, under the condition of different receivers $R_{i}$, $i=1,\cdots ,N_{R}$.

### 5.2. Compressed Sensing Model for an Individual Receiver

At the *i*-th receiver $R_{i}$, the received measurement signal can be represented via the grids in target spate space, as:

(14) $$r_{k}^{i}\left(t\right)=\sum_{l=1}^{N_{g}}\left\{\alpha_{k}^{i,l}\cdot \sum_{n=1}^{W}p\left(t-\left(n-1\right)\varepsilon -\left(k-1\right)\Delta T-\tau_{k}^{i,l}\right)\cdot e^{j2\pi f_{k}^{i,l}\cdot t}\right\}+w_{k}^{i}\left(t\right),$$

where $\alpha_{k}^{i,l}$ denotes the reflection coefficient corresponding to the *l*-th grid between the transmitter and the *i*-th receiver; $\tau_{k}^{i,l}$ is the *l*-th grid originated delay, and $f_{k}^{i,l}$ is the Doppler shift frequency of the *l*-th grid, both measured by the *i*-th receiver; $w_{k}^{i}\left(t\right)$ is the complex envelope of the overall disturbance at the *i*-th receiver.

The state vector corresponding to the *l*-th grid, $g_{k}^{l}$, contributes to the received signal if it is occupied by a target. We define $\varphi_{k}^{i,l}\left(t\right)$ as the *l*-th grid’s contribution to the received signal, as:

(15) $$\varphi_{k}^{i,l}\left(t\right)=\sum_{n=1}^{W}p\left(t-\left(n-1\right)\varepsilon -\left(k-1\right)\Delta T-\tau_{k}^{i,l}\right)e^{j2\pi f_{k}^{i,l}\cdot t}.$$

At the *i*-th receiver, a sequence of discrete outputs of the received signal is sampled, which forms a measurement vector $y_{k}^{i}$, as:

(16) $$y_{k}^{i}={\left[r_{k}^{i}\left(1\right),r_{k}^{i}\left(2\right),\cdots ,r_{k}^{i}\left(W\right)\right]}^{T},$$

where $r_{k}^{i}\left(n\right),n=1,\cdots ,W$ are discrete output samples collected by the *i*-th receiver, at scan *k*.

The measurement vector $y_{k}^{i}$ can be represented in a compressed sensing framework, as in (17),

(17) $$y_{k}^{i}=\Phi_{k}^{i}\xi_{k}^{i}+e_{k}^{i},$$

where $\Phi_{k}^{i}$ is a sensing matrix and $\xi_{k}^{i}$ is a sparse grid reflection vector at the *i*-th receiver. We have:

(18) $$\Phi_{k}^{i}=\left[\varphi_{k}^{i,1}\cdots \varphi_{k}^{i,l}\cdots \varphi_{k}^{i,N_{g}}\right],$$

where $\varphi_{k}^{i,l}$ is the *l*-th column of the sensing matrix, i.e., $\varphi_{k}^{i,l}={\left[\varphi_{k}^{i,l}\left(1\right),\varphi_{k}^{i,l}\left(2\right),\cdots ,\varphi_{k}^{i,l}\left(W\right)\right]}^{T}$. It can be seen from (15) that $\varphi_{k}^{i,l}$ is the *l*-th grid’s contribution to the received signal and is deterministic in the condition of fixed grids. Since the division of the state space with grids is assumed fixed prior in this work, the sensing matrix is deterministic in nature.

### 5.3. General JSM-1 in a Multistatic Radar System

In the multistatic radar system, assume that the *i*-th receiver $R_{i}$ has bi-directional communication links with a number of neighboring nodes (receivers), which are denoted as $U_{i}^{j},j=1,\cdots ,{NE}_{i},$ $i\;=\;1,\cdots ,N_{R}$, where ${NE}_{i}$ denotes the number of neighboring nodes of the *i*-th receiver. For each neighboring node $U_{i}^{j}$, we can obtain a standard equation in compressed sensing, as:

(19) $$y_{k}^{j}=\Phi_{k}^{j}\xi_{k}^{j}+e_{k}^{j},\;\;j=1,\cdots ,{NE}_{i},$$

where $y_{k}^{j}$, $\Phi_{k}^{j}$, $\xi_{k}^{j}$ and $e_{k}^{j}$ denote the measurement vector, sensing matrix, sparse grid reflection vector and noise vector, respectively, for the *j*-th neighboring node of the *i*-th receiver.

It is assumed that the *i*-th receiver and its ${NE}_{i}$ neighboring nodes observe a surveillance area. For each individual receiver, it cannot “see” all of the targets at a time. This is reasonable in practice considering that the receivers are located at different positions with different viewing angles, and each receiver has a different detection probability (less than one). Therefore, some targets are observed by all of the receivers, namely common targets; and the others are observed by individual receiver, which are named as innovation targets.

The common targets’ states are the same as different observers (receivers) located at different positions, when the targets’ states and receivers’ positions are defined in the same coordinate system. Thus, for different receivers, their corresponding sparse grid reflection vectors share the same locations of part of the nonzero reflection coefficients (corresponding to the common targets), i.e., having the same common support set. However, the reflection coefficients of a common target observed by different receivers are not the same due to different parameters of each individual receiver; thus, the common components of different sparse grid reflection vectors are different, which does not fit the standard JSM-1 (2) presented in Section 2.

A general JSM-1 is adopted in this work, which relies only on Equation (3), focusing on common support set. In this condition, it is assumed that different sparse grid reflection vectors share a common support set, while having different reflection coefficients. Since the proposed DCSMP algorithm focuses on support set estimation instead of estimating the common component (the reflection coefficients), the proposed DCSMP algorithm can cope with the general JSM-1 efficiently.

### 5.4. Simulation Environment Setup

A multistatic radar system consisting of a transmitter and three receivers are considered in this simulation example. The transmitter is located at $\mathrm{tr}={\left[0,0,0\right]}^{T}$ km; the receivers are located at $r_{1}\;=\;{\left[0,8,0\right]}^{T}$ km, $r_{2}={\left[8,0,0\right]}^{T}$ km and $r_{3}={\left[8,8,0\right]}^{T}$ km, respectively, in a 3D Cartesian coordinate system. The three receivers are connected with bi-directional communication links. The carrier frequency ($f_{c}$) for each receiver is 10 GHz. The number of discrete samples collected at each receiver (*W*) is 80. The volume of the surveillance position space (represented in $px_{k},py_{k},pz_{k}$) is $10^{3}\;\times \;10^{3}\times \;10^{3}$ m${}^{3}$, which is divided into 10×10×10 grid points; the volume of the surveillance velocity space (represented in $vx_{k},vy_{k},vz_{k}$) is 60×60×60 (m/s)${}^{3}$, which is divided into 2×2×2 grid points. Therefore, the total number of grids in state space ($N_{g}$) is $8\times 10^{3}$.

### 5.5. Identification of Multiple Targets’ States

The proposed DCSMP algorithm is used to identify multiple targets (including common and innovation targets) in the state space. The achievable resolution of the sparse vector in state space obtained by the DCSMP algorithm is evaluated and compared with the DIPP algorithm. We focus on the difference in position while assuming that all of the targets have the same velocity, for clarity in representation.

Three cases are considered in the simulation example: (a) separately-distributed targets; (b) closely-spaced common targets; (c) closely-spaced innovation targets observed by the receiver R1. The addressed scenarios are characterized by the signal-to-noise ratio (SNR), which is set to 20 dB. The simulation parameters for the three cases are listed in Table 3, Table 4 and Table 5.

**Figure 3:**
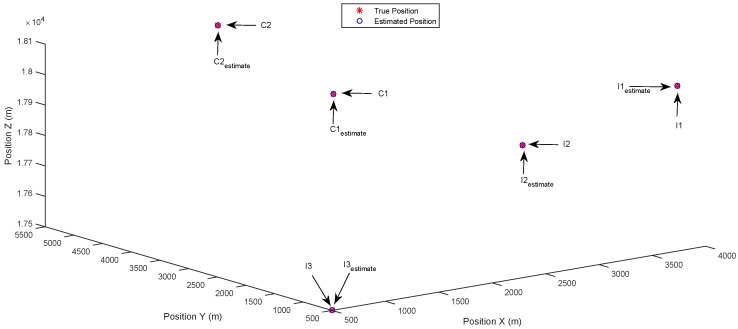
Case 1: Separately-distributed targets. Estimated positions in 3D space using the DCSMP algorithm.

**Figure 4:**
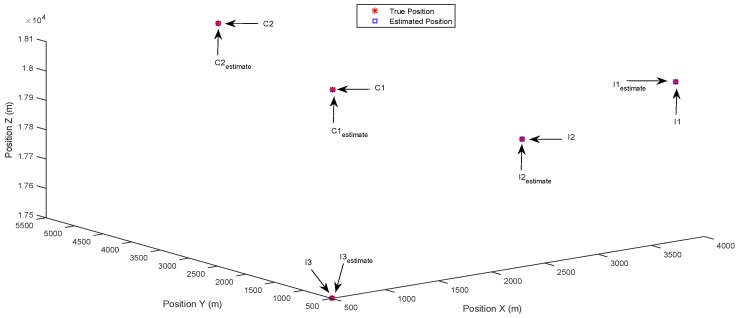
Case 1: Separately-distributed targets. Estimated positions in 3D space using the distributed parallel pursuit (DIPP) algorithm.

**Figure 5:**
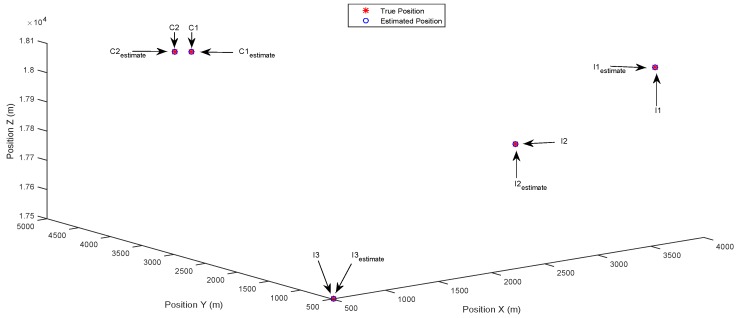
Case 2: Closely-spaced common targets. Estimated positions in 3D space using the DCSMP algorithm.

**Figure 6:**
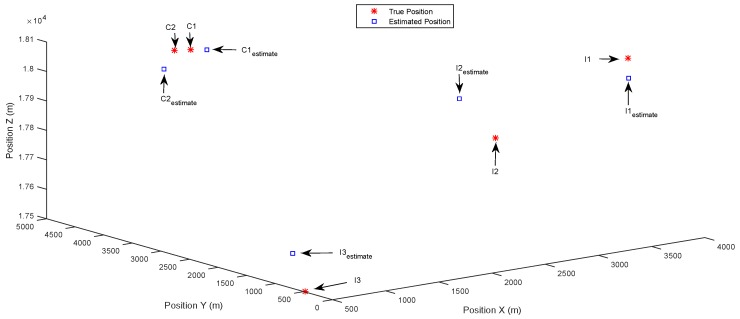
Case 2: Closely-spaced common targets. Estimated positions in 3D space using the DIPP algorithm.

**Figure 7:**
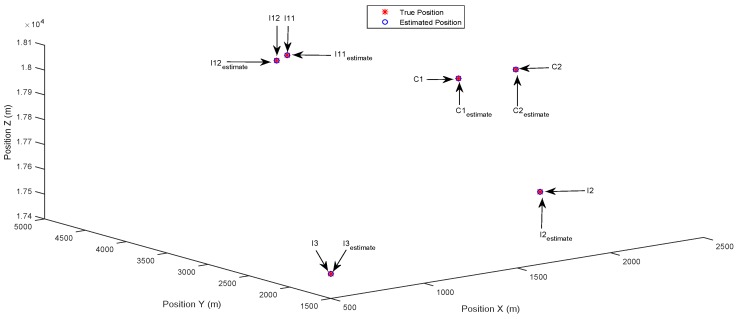
Case 3: Closely-spaced innovation targets observed by receiver R1. Estimated positions in 3D space using the DCSMP algorithm.

**Figure 8:**
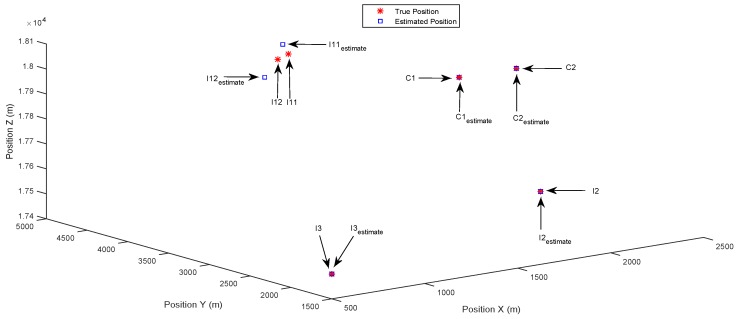
Case 3: Closely-spaced innovation targets observed by R1. Estimated positions in 3D space using the DIPP algorithm.

The simulation results are shown in Figure 3, Figure 4, Figure 5, Figure 6, Figure 7 and Figure 8. The estimated positions shown in each figure are the combination of the results from three receivers, including the common targets observed by all of the receivers and the innovation targets observed by the individual receiver. This is reasonable since in practice, at the final stage of estimation, each receiver will send its estimated locations of the innovation targets to its neighboring nodes, and all of the connected receivers will have a common scene of the surveillance area.

#### 5.5.1. Separately Distributed Targets

Figure 3 and Figure 4 show the results of the identification of multiple separately-distributed targets in the state space, using the DCSMP and DIPP algorithm, respectively. In Figure 3, the true positions of targets are denoted as stars (“*”), with text indications “C1” and “C2” for common targets and “I1”, “I2”, “I3” for innovation targets. The estimated positions of targets are denoted as circles (“o”), with text indications “$C1_{estimate}$” and “$C2_{estimate}$” for estimated common targets and “$I1_{estimate}$”, “$I2_{estimate}$”, “$I3_{estimate}$” for estimated innovation targets. It can be seen from Figure 3 that the multiple separately-distributed targets (including both the common and innovation targets) are accurately identified in the state space using the DCSMP algorithm, and similar results appear in Figure 4, for the DIPP algorithm.

#### 5.5.2. Closely-Spaced Common Targets

The main challenge of this scenario arises from the small separation between two closely-spaced common targets C1 and C2. Figure 6 shows that the DIPP algorithm fails in distinguishing C1 and C2. This is due to the reason that the DIPP algorithm cannot efficiently cope with the sensing matrix with high coherence due to the high resolution of the state space. Moreover, since the estimated common support set (i.e., estimated positions of the common targets) is adopted as the side information to calculate the innovation support set by the DIPP algorithm, the wrongly-estimated common support set leads to failure in estimating the innovation support set, for each receiver. Therefore, the positions of the innovation targets cannot be identified accurately (Figure 6).

In Figure 5, the common targets are accurately identified, which verifies that the proposed DCSMP algorithm is capable of dealing with the sensing matrix with high coherence. As a consequence, the innovation targets are accurately identified based on the correctly-estimated common support set.

#### 5.5.3. Closely-Spaced Innovation Targets

The main challenge in this scenario arises from the small separation between two closely-spaced innovation targets I11 and I12, observed by the receiver R1. Figure 8 shows that the DIPP algorithm succeeds in estimating the positions of two separately-distributed common targets. The estimated common support set is then input as the side information to calculate the innovation support set, at three receivers, respectively. Figure 8 shows that the first receiver R1 fails in distinguishing the two closely-spaced innovation targets I11 and I12, while the other two receivers R2 and R3 succeed in identifying their corresponding innovation targets I2 and I3, respectively. In Figure 7, it can be seen that the proposed DCSMP algorithm succeeds in identifying both the common and innovation targets.

### 5.6. Random Testing Cases

A sequence of random cases is tested where 20 targets (including common and innovation targets) are randomly distributed in the three-dimensional position space. Five hundred Monte Carlo simulations are performed for each trail. Considering that each individual receiver cannot “see” all of the targets at a time, the numbers of common and innovation targets are set as 15 and 5, respectively. Thus, we have the following parameter setup for the simulation: the signal dimensionality $N\;=\;8\times \;10^{3}$, the measurement dimensionality M=80, the sparsity level K=20, the common support set J=15, the innovation support set $I_{p}=5$, the number of all nodes (receivers) $N_{R}=3$ and the number of Monte Carlo trails $N_{MC}=500$.

Two metrics are utilized to evaluate the performance of the proposed algorithm. The first metric is called distributed reconstruction error (DRE), which is defined as:

(20) $$DRE=\frac{1}{|N_{R}|}\Sigma_{p=1}^{|N_{R}|}\left[\frac{1}{N_{MC}}\Sigma_{i=1}^{N_{MC}}\frac{\left|\right|{\hat{x}}_{p}^{i}-x_{p}^{i}{\left|\right|}_{2}}{\left|\right|x_{p}^{i}{\left|\right|}_{2}}\right],$$

where $x_{p}^{i}$ represents the true signal for the *p*-th receiver in the *i*-th Monte Carlo trail and ${\hat{x}}_{p}^{i}$ represents the estimated signal. Our objective is to achieve a lower DRE considering the whole decentralized network. We also adopt the average support-set cardinality error (ASCE) as a direct evaluation of the support-set recovery performance [12]. Note that the ASCE has the range [0,1], and our objective is to achieve a lower ASCE.

We now provide the average performance results using the performance measures DRE and ASCE, which are shown in Table 6. The performance of OMP and SP algorithms is included in the simulation as a benchmark characterizing a single-sensor (disconnected) scenario. From Table 6, it can be seen that the proposed DCSMP algorithm obtains the lowest DRE and ASCE, which verifies that the proposed DCSMP algorithm outperforms the three other algorithms (DIPP, SP and OMP), in reconstructing the sparse vector with high resolution, in a decentralized network.

### 5.7. Simulation Results on a Large-Scale Sensor Network

**Figure 9:**
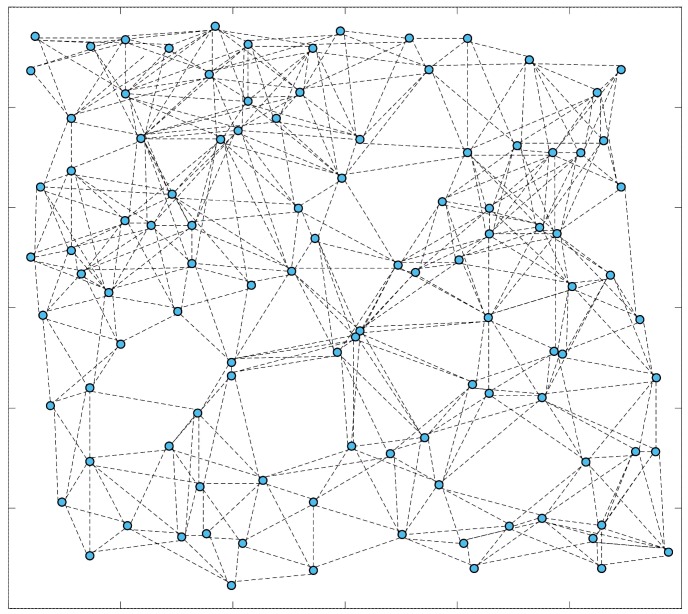
The connectivity topology of the tested network comprised of 105 nodes.

This simulation is to validate the performance of the proposed DCSMP algorithm on a large-scale sensor network. The network topology is built along the random geometric graph model, presented by Penrose in [23], where a number of nodes are randomly distributed in an area. Each node connects with its neighbor nodes located within a certain distance from it. Figure 9 shows a typical setup of such a network consisting of 105 nodes. The proposed DCSMP algorithm is compared to the compressed sensing-based algorithms, e.g., the SP, OMP and DIPP algorithms, as well as non compressed sensing based algorithms, e.g., principal component analysis (PCA) [24] and the distributed wavelet compression (DWC) algorithm [25].

The signal model is constructed based on the multistatic radar system. The sparse grid reflection vector is chosen as the original signal. For the conventional compressed sensing-based algorithms, e.g., the SP and OMP algorithm, the measurements are compressed by a down-sampling measurement matrix before transmitting to the neighboring nodes. For the non-compressed sensing-based approaches, e.g., the PCA and DWC algorithm, the data compression is achieved after aggravating signals from neighboring nodes. For the above two kinds of algorithms, the measurements are transmitted in the network. In contrast, in the DCSMP and DDIP algorithm, only the estimated candidate support sets are transmitted between nodes in the network, which significantly reduces the transmission burden.

**Figure 10:**
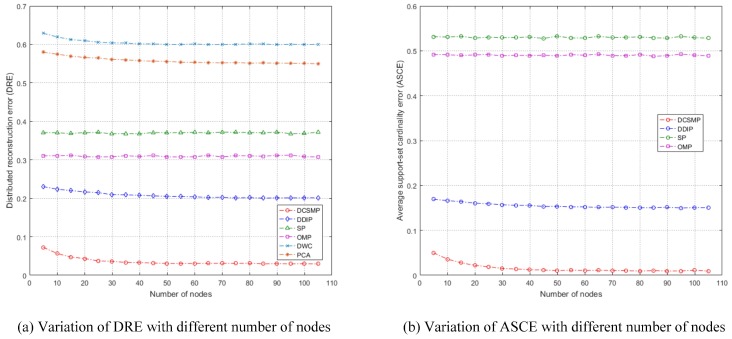
Performance comparison of different algorithms with a varying number of nodes. (**a**) Variation of DRE with different numbers of nodes; (**b**) variation of ASCE with different numbers of nodes.

A sequence of random cases is tested where a number of targets (including common and innovation targets) are randomly distributed in the three-dimensional position space. The simulation parameters are the same as those in Section 5.6. Two metrics, DRE and ASCE, are utilized to evaluate the reconstruction performance of different algorithms. Figure 10a shows the variation of DRE with different number of nodes, for different algorithms. It can be seen from Figure 10a that the proposed DCSMP algorithm achieves the lowest DRE. The non-compressed sensing-based algorithms (e.g., PCA and DWC) cannot reconstruct the original signal perfectly at a down-sampling rate, thus they achieve large DREs. Though the conventional compressed sensing algorithms (e.g., SP and OMP) and DIPP algorithm can reconstruct the original signal accurately at a down-sampling rate, they cannot cope with the sensing matrix with high coherence, thus achieving moderate DREs, compared with the proposed DCSMP algorithm. Moreover, it can be seen from Figure 10a that the DREs of the two algorithms, DCSMP and DDIP, slightly decrease with the increase of network size. This is due to the reason that the information (the candidate support set or the measurements) received by each node increases with the size of the network expands, thus resulting in a more accurate estimated signal. Figure 10b shows the variation of ASCE with different numbers of nodes. The proposed DCSMP algorithm achieves the lowest ASCE. This verifies that the proposed DCSMP algorithm outperforms the three other algorithms (DIPP, SP and OMP) in dealing with the sensing matrix with high coherence.

## 6. Conclusions

A novel DCSMP algorithm is proposed to tackle the JSM-1 in a decentralized scenario. The proposed algorithm adopts deterministic sensing matrices built directly on the real acquisition systems. In the proposed algorithm, the process of estimating the common support set is to decouple that of estimating the innovation support set. Thus, the inaccurately estimated common support set will have no impact on estimating the innovation support set. The simulation results show that the proposed algorithm can perform successful sparse recovery in a decentralized manner across various receivers, in a multistatic radar system.

## Figures and Tables

**Table 1:** Complexity analysis for online processing.

Rough Estimation	Data Fusion	Innovation Support Set Estimation	Final Estimation
O($MD_{p}$)	Negligible	O($|\hat{J}|\cdot M$)+O($M\cdot |{\hat{J}}^{c}|$)	$N_{co}\cdot O\left(KM\right)$

**Table 2:** Computational complexity of the DCSMP Algorithm.

Offline Processing	Online Processing
O$\left(\frac{N(N-1)}{2}M\right)$	O($MD_{p}$) + O($|\hat{J}|\cdot M$)+O($M\cdot |{\hat{J}}^{c}|$) + $N_{co}\cdot O\left(KM\right)$

**Table 3:** Case 1: Separately-distributed targets.

Target Number	Reflected Amplitude (dB)	Positions (m)	Observer
C1	2	${\left[1800,2900,18000\right]}^{T}$	R1,R2,R3
C2	6	${\left[1900,5100,18100\right]}^{T}$	R1,R2,R3
I1	13	${\left[4000,1000,18000\right]}^{T}$	R1
I2	2	${\left[3500,900,17900\right]}^{T}$	R2
I3	6	${\left[500,500,17500\right]}^{T}$	R3

**Table 4:** Case 2: Closely-spaced common targets.

Target Number	Reflected Amplitude (dB)	Positions (m)	Observer
C1	2	${\left[1800,4900,18000\right]}^{T}$	R1,R2,R3
C2	6	${\left[1700,5000,18000\right]}^{T}$	R1,R2,R3
I1	13	${\left[3600,600,18100\right]}^{T}$	R1
I2	2	${\left[3000,1800,17800\right]}^{T}$	R2
I3	6	${\left[510,500,17500\right]}^{T}$	R3

**Table 5:** Case 3: Closely-spaced innovation targets observed by R1.

Target Number	Reflected Amplitude (dB)	Positions (m)	Observer
C1	2	${\left[1800,4900,18000\right]}^{T}$	R1,R2,R3
C2	6	${\left[1800,2200,18100\right]}^{T}$	R1,R2,R3
I11	13	${\left[1800,5000,17900\right]}^{T}$	R1
I12	13	${\left[1700,4900,17900\right]}^{T}$	R1
I2	2	${\left[2500,1500,17400\right]}^{T}$	R2
I3	6	${\left[500,1500,17500\right]}^{T}$	R3

**Table 6:** Performance results. DRE, distributed reconstruction error; ASCE, average support-set cardinality error.

Algorithm	DRE	ASCE
DCSMP	0.07	0.05
DIPP	0.23	0.17
SP	0.37	0.53
OMP	0.31	0.49

## References

[1] Lin L., Liu F., Jiao L. (2014). Compressed Sensing by Collaborative Reconstruction on Overcomplete Dictionary. Signal Process..

[2] Wang J.C., Lee Y.S., Lin C.H., Wang S.F., Shih C.H., Wu C.H. (2016). Compressive Sensing-Based Speech Enhancement. IEEE/ACM Trans. Audio Speech Lang. Process..

[3] Wang D., Wan J., Chen J. (2016). An Online Dictionary Learning-Based Compressive Data Gathering Algorithm in Wireless Sensor Networks. Sensors.

[4] Yin J., Yang Y., Wang L. (2016). An Adaptive Data Gathering Scheme for Multi-Hop Wireless Sensor Networks Based on Compressed Sensing and Network Coding. Sensors.

[5] Bu H., Tao R., Bai X. (2016). Regularized Smoothed *l*^0^ Norm Algorithm and its Application to CS-based Radar Imaging. Signal Process..

[6] Baron D., Wakin M., Duarte M., Sarvotham S., Baraniuk R. Distributed compressed sensing. http://ai2-s2-pdfs.s3.amazonaws.com/6388/e944d5929cb6490792429085ed31c0ad776b.pdf.

[7] Zhang Q., Fu Y., Li H., Rong R. (2014). Optimised projections for generalised distributed compressed sensing. Electron. Lett..

[8] Zeng F., Li C., Tian Z. (2011). Distributed compressive spectrum sensing in cooperative multihop cognitive networks. IEEE Trans. Signal Process..

[9] Tan L., Wu M. (2016). Data reduction in wireless sensor networks: A hierarchical LMS prediction approach. IEEE Sens. J..

[10] Wu M., Tan L., Xiong N. (2016). Data prediction, compression, and recovery in clustered wireless sensor networks for environmental monitoring applications. Inf. Sci..

[11] Wu M., Tan L., Xiong N. (2015). A Structure Fidelity Approach for Big Data Collection in Wireless Sensor Networks. Sensors.

[12] Sundman D., Chatterjee S., Skoglund M. (2016). Design and Analysis of a Greedy Pursuit for Distributed Compressed Sensing. IEEE Trans. Signal Process..

[13] Baron D., Duarte M.F., Sarvotham S., Wakin M.B., Baraniuk R.G. An information theoretic approach to distributed compressed sensing. Proceedings of the 43rd Allerton Conference Communication Control, and Computing.

[14] Schnelle S.R., Laska J.N., Hegde C., Duarte M.F., Davenport M.A., Baraniuk R.G. Texas hold ’Em algorithms for distributed compressive sensing. Proceedings of the IEEE International Conference Acoustics, Speech and Signal Processing (ICASSP).

[15] Coluccia G., Magli E., Roumy A., Toto-Zarasoa V. Lossy compression of distributed sparse sources: A practical scheme. Proceedings of the 2011 19th European Signal Processing Conference (EUSIPCO 11).

[16] Valsesia D., Coluccia G., Magli E. Joint recovery algorithms using difference of innovations for distributed compressed sensing. Proceedings of the 2013 Asilomar Conference on Signals, Systems and Computers.

[17] Matamoros J., Fosson S.M., Magli E., Antón-Haro C. (2015). Distributed ADMM for In-Network Reconstruction of Sparse Signals With Innovations. IEEE Trans. Signal Inf. Process. Netw..

[18] Liu J., Mallick M., Han C.Z., Yao X.H., Lian F. (2014). Similar sensing matrix pursuit: An efficient reconstruction algorithm to cope with deterministic sensing matrix. Signal Process..

[19] Liu J., Mallick M., Lian F., Han C.Z., Sheng M.X., Yao X.H. (2015). General similar sensing matrix pursuit: An efficient and rigorous reconstruction algorithm to cope with deterministic sensing matrix with high coherence. Signal Process..

[20] Foucart S., Rauhut H. (2013). A Mathematical Introduction to Compressive Sensing.

[21] Needell D., Tropp J.A. (2009). COSAMP: Iterative signal recovery from incomplete and inaccurate samples. Appl. Comput. Harmonic Anal..

[22] Chen W., Rodrigues M.R.D., Wassell I.J. (2012). A Frechet Mean Approach for Compressive Sensing Date Acquisition and Reconstruction in Wireless Sensor Networks. IEEE Trans. Wirel. Commun..

[23] Penrose M. (2004). Random Geometric Graphs.

[24] Chen F., Wen F., Jia H. Algorithm of Data Compression Based on Multiple Principal Component Analysis over the WSN. Proceedings of the 6th International Conference on Wireless Communications Networking and Mobile Computing (WiCOM).

[25] Ciancio A., Pattem S., Ortega A., Krishnamachari B. Energy-efficient data representation and routing for wireless sensor networks based on a distributed wavelet compression algorithm. Proceedings of the Fifth International Conference on Information Processing in Sensor Networks (IPSN 2006).

